# The Faith, Activity, and Nutrition (FAN) Dissemination and Implementation Study: 24-Month Organizational Maintenance in a Countywide Initiative

**DOI:** 10.3389/fpubh.2020.00171

**Published:** 2020-05-14

**Authors:** Sara Wilcox, Ruth P. Saunders, Danielle Jake-Schoffman, Brent Hutto

**Affiliations:** ^1^Prevention Research Center, Arnold School of Public Health, University of South Carolina, Columbia, SC, United States; ^2^Department of Exercise Science, Arnold School of Public Health, University of South Carolina, Columbia, SC, United States; ^3^Department of Health Promotion, Education, and Behavior, Arnold School of Public Health, University of South Carolina, Columbia, SC, United States; ^4^Department of Health Education and Behavior, College of Health and Human Performance, University of Florida, Gainesville, FL, United States

**Keywords:** RE-AIM, physical activity, nutrition, community, faith-based, maintenance, sustainability

## Abstract

**Introduction:** Despite the important role that faith-based organizations can play in eliminating health disparities, few studies have focused on organizational change and maintenance of interventions in this setting, making their long-term impact unknown. This study reports 24-month maintenance of the Faith, Activity, and Nutrition (FAN) program in a southeastern county. Previously reported findings of reach, adoption, implementation, and effectiveness are also summarized.

**Methods:** Church coordinators from 35 intervention churches (97% predominantly African American) located in a rural, medically underserved county in South Carolina were interviewed at baseline (2015), and 12- and 24-months post-training regarding implementation of physical activity (PA) and healthy eating (HE) components of the FAN program. Guided by the RE-AIM framework, organizational maintenance was defined as church coordinator-reported 24-month implementation of the four FAN components (providing opportunities, setting guidelines/policies, sharing messages, engaging pastor). Repeated measures analyses (mixed models) examined change in implementation over time. Churches were also classified as maintainers, non-sustained implementers, and low implementers for each FAN component. Statistical analyses were conducted in 2019.

**Results:** Church coordinators reported significantly greater implementation of both PA and HE FAN components at 12 and 24 months compared to baseline (medium to large effects). The percentage of churches classified as maintainers ranged from 21 to 42 and 27 to 94% across PA and HE components, respectively. Most churches (58% for PA, 97% for HE) were maintaining at least one FAN component at 24 months.

**Conclusions:** These promising findings position FAN well for the national implementation study now underway.

**Trial Registration:** This study is registered at www.clinicaltrials.gov NCT02868866.

## Introduction

Most U.S. adults (70.6%) report a Christian affiliation, and around 36% report attendance at religious services at least once per week (1, 2). Faith-based settings are a viable setting for health promotion efforts, but a number of gaps in the literature limit the ability to scale-up programs for wider dissemination. For example, only 9% of studies in a recent review reported implementation fidelity (3), and studies examining the sustainability or maintenance of intervention effects are rare. Furthermore, most interventions focus on individual rather than organizational (church) level outcomes. Faith, Activity, and Nutrition (FAN) is an evidence-based program designed to help churches make policy, systems, and environmental change to support physical activity (PA) and healthy eating (HE). Based on its significant impact on improving church attendees' PA and dietary change, FAN is indexed in the National Cancer Institute's Research Tested Intervention Programs (4) and is cited as a promising intervention in the Rural Health Information Hub (5). Most recently we undertook the FAN dissemination and implementation (D&I) study in two phases. In the first phase of the FAN D&I study, we partnered with a county coalition to offer FAN to all churches in a county in South Carolina. In the second phase of the FAN D&I study, we partnered with a large religious denomination and offered FAN to all churches of that denomination in the state. Phase 1 is the focus of this paper.

The FAN D&I study was guided by the RE-AIM framework (6). Phase 1 of the study included an examination of each component of the framework. The primary goal of this paper is to report the 24-month maintenance of FAN in intervention churches participating in Phase 1. A secondary goal of the paper is to summarize previously published findings from the other RE-AIM components in Phase 1—reach, effectiveness, adoption, and implementation—so that readers have a full understanding of how the full RE-AIM framework was applied in this study and the major findings. The reader is referred to previous papers for more details regarding adoption, reach, effectiveness, and 12-month implementation of FAN; (7, 8) recruitment, training, and implementation of the trainings and technical assistance by community health advisors; (9) and barriers and facilitators to 12-month implementation (10).

## Materials and Methods

### Design

Phase 1 of the FAN D&I study was a group-randomized trial and an academic-community partnership (7). The study was conducted in Fairfield County, South Carolina (23,956 residents) (11), a medically underserved and health professional shortage area (12). A high proportion of residents are Black/African American (59.1%), and 21.2% of residents live in poverty (11, 13).

All churches (*N* = 132) in the county were invited to participate in the study. Enrolled churches (*N* = 59) were randomized to either an intervention (*n* = 39) or control (delayed intervention, *n* = 20) condition. The delayed intervention control churches were trained 12-months after intervention churches were trained and after effectiveness measurements were taken. This delayed treatment design allowed us to compare intervention to control churches on 12-month implementation and effectiveness. This design was also deemed acceptable by the community who viewed 1 year as a realistic time to wait for the full training. However, because the delayed intervention control group was trained and had 1 year of implementation at the 24-month follow up assessment, it was not possible to compare intervention to true control churches at 24 months. As a result, this paper examines whether the implementation outcomes seen at 12 months, which were significantly different than measurements taken at 12 months in control churches, were maintained in the intervention churches at the 24-month assessment. As reported elsewhere (7), 97% of intervention churches had predominantly Black/African American members, 42% had <50 regular attendees, and the most common religious denominations were Baptist (46%), non-denominational or independent (26%), AME/AME Zion (11%), and Pentecostal (8%).

### FAN Intervention

FAN is an evidence-based program that helps churches to create policy, systems, and environmental changes to support increased PA and HE in members. It was developed using a community-based participatory research approach in which church leaders, church lay representatives, and university staff and faculty collaborated to develop, implement, and evaluate the program (14). Guided by Cohen's structural model of health behavior (15), FAN's four structural components are to provide opportunities, set guidelines (policies), engage pastors, and share messages for PA and HE. As described elsewhere (7, 8), the university collaborated with community organizations in Fairfield County, SC to identify and train community health advisors who, in turn, delivered trainings, and provided technical assistance to churches (9).

Each participating church formed a committee, led by a church coordinator (liaison with the research staff and responsible for coordinating the implementation efforts in the church). Church committees attended a 1 day training where they were guided through an active “assessment and planning” process that was organized according to the four structural components of FAN. While there was a set of activities that all churches were asked to implement (i.e., distribute bulletin inserts or handouts, share messages during worship services about PA and HE, distribute educational materials, create a FAN bulletin board to display PA and HE materials to congregants, and suggest guidelines/policies that the pastor could set), churches had flexibility to choose specific activities within each of the structural components so that the activities matched the culture, norms, and preferences of their congregations. Each church committee created and submitted a plan and budget for how program components would be implemented in their church (this plan was started during the in-person training), and implemented the program in their church over the next 12 months with technical assistance from a community health advisor. Trainings, technical assistance, and program materials emphasized the scriptural relevance of physical health from a Christian tradition without reference to specific denominations or doctrines.

Community health advisors provided brief monthly telephone support for 12 months after training (4 calls to the pastor, 8 calls to the church coordinator). Research staff also emailed the pastor and church coordinator monthly program materials, provided at training, as a reminder to use them. Near the end of the first year of the study, the community health advisor encouraged churches to create a revised plan for implementing FAN activities in the upcoming year. The core set of activities described previously (e.g., distributing bulletin inserts or handouts) remained the same, but consistent with the underlying philosophy and approach of FAN, and consistent with the assessment and planning process used during training, churches were encouraged to assess what was working well, what could be improved, and what was not working with regard to increasing opportunities, messages, pastor support, and setting guidelines (policies) for PA and HE, and to make necessary adjustments to meet these goals and also keep their activities fresh and engaging for members. This consistency over time in approach and intervention components and goals ensured that churches adhered to the essential program elements. During the second year of the program, research staff emailed the pastor and church coordinator once per month with new materials to share with their congregations (a bulletin insert that tied a health message to Scripture, educational materials, and a website).

### Data Collection Procedures

We collected implementation data from church coordinators, rather than conducting on-site observations, for two main reasons. First, the logistics of collecting data on-site for such a large number of participating churches were prohibitive. Second, components of our ecological intervention were meant to be embedded before, during, and after church events and meetings, making it very difficult to capture the range of activities over a period of even a week during an on-site observation. It is noteworthy that member reports of the church environment were quite consistent with reports from church coordinators in our examination of 12-month implementation (7, 8), making us confident in the validity of church coordinator reports.

Baseline, 12, and 24-month telephone interviews with church coordinators were conducted by the Survey Research Laboratory at the University of South Carolina using a computer-aided telephone interviewing system. Interviewers received specialized training for this study prior to data collection. Interviews were conducted from September 2 to October 28, 2015 at baseline; September 6 to November 3, 2016 at 12 months; and September 21 to December 19, 2017 at 24 months. Three out of 39 intervention churches did not attend training and one additional intervention church withdrew after training. Twelve-month interviews were conducted with 35 (89.7%) church coordinators. Twenty-four-month interviews were conducted with 33 (84.6%) church coordinators. At 24 months, three of these were completed via a paper-and-pencil survey because the church coordinators were not available for a telephone interview.

### Measures

Implementation measures of the FAN components (15) were based on the guiding conceptual model (8) and were adapted from the implementation measures used in our prior FAN study (16). All measures were reviewed by community partners to ensure clarity and acceptability. PA implementation was assessed with 10 items-−1 for guidelines (policies), 4 for opportunities (2 focused on integrating PA into existing church events, 1 on offering PA programs, and 1 on sharing information about free or low-cost PA opportunities in the community), 1 for pastor support (sharing messages during services), and 4 for messages (church bulletins, bulletin board, person other than pastor sharing messages during services, sharing messages at church meetings, and events). HE implementation was assessed with 9 items-−2 for guidelines (policies) (fruit and vegetables), 2 for opportunities (fruit and vegetables), 1 for pastor support (sharing messages during services), and 4 for messages (same channels as described for PA). Mean scores were calculated for multi-item scales, and composite scores for PA and HE were computed, each representing the average of the four components. Each item was rated on a 4-point Likert scale, where, depending on the question, 1 indicated “rarely or never” or “not at all,” 2 indicated “very little” or “every few months,” 3 indicated “some of the time” or “about monthly,” and 4 indicated “almost all of the time” or “about weekly.” For the guideline (policy) questions, a score of 3 indicated that the guideline was partially in place whereas a 4 indicated it was fully in place.

The criteria for evidence of acceptable implementation (12 months) was set a priori at 3 or 4 out of 4. Although we did not set an a priori criteria of acceptable implementation for maintenance (24 months), for the current analysis we use the same level of evidence as we did for 12 months (i.e., 3 or 4 out of 4).

For each of the four components, churches were categorized as maintainers if they met the criteria for maintenance at 24 months, non-sustained implementers if they met the criteria for implementation at 12 months but were below the criteria for maintenance at 24 months, and low implementers if they were below the criteria for implementation at 12 months and below the criteria for maintenance at 24 months.

### Data Analyses

We tested differences in implementation scores among early intervention churches over time with repeated measures regression models using mixed linear models (SAS PROC MIXED). When the time effect was significant, we examined pairwise least square mean differences from baseline to 12 months, baseline to 24 months, and 12 to 24 months. We calculated effect sizes (Cohen's *d*) from baseline to 12 months and baseline to 24 months. We also categorized the pattern of meeting implementation and maintenance criteria for each church over time, and described the proportion of churches classified as maintainers, non-sustained implementers, and low implementers for each intervention component. Finally, we reported the percentage of churches maintaining four, three, two, one, or zero of the structural components of FAN for PA and HE, and the percentage of churches who either maintained or improved relative to baseline for four, three, two, one, or zero of these components.

## Results

### Reach, Adoption, Implementation, and Effectiveness

Phase 1 reach, adoption, implementation, and effectiveness results have been published in prior papers. The results are summarized here so that readers have a fuller understanding of how the full RE-AIM framework was applied in this study and the key findings.

#### Reach and Adoption

All 132 churches in the county were invited to participate in the study. As reported previously (7), FAN was adopted by 42% of these churches and reached at least 42% of regular church attendees and at least 15% of residents in the county. Churches with predominantly black/African American congregations and those who participated in an earlier tobacco-free county initiative were significantly more likely to adopt FAN. Church size and church denomination were not related to adoption. When compared to county-level data, the sample of church attendees from adopting churches were more likely to be 65 years of age or older, obese, women, and African American.

#### Implementation

Implementation in this study was studied at two levels. First, we studied the fidelity of delivering the intervention to the church committees (9). Second, we studied the degree to which church committees implemented the intervention (FAN) as intended in their churches (7, 8).

Three community health advisors were recruited and trained to deliver the church committee trainings and technical assistance calls to church coordinators and pastors, and a paper describing this process and findings is available (9). One community health advisor resigned prior to implementing any of the duties due to unforeseen scheduling conflicts. The remaining two community health advisors trained 142 church committee members from 36 intervention churches and 60 church committee members from 18 control churches. In the post-training evaluation, church committees positively rated how well the training prepared them to put the program into place. University staff who observed the trainings rated nearly complete coverage of all content areas and rated factors such as the community health advisors' ability to engage participants very positively. A high percentage of calls (>90%) were delivered, and calls averaged around 7 min in duration (9). Thus, fidelity to delivering the intervention was high.

Two papers have reported the churches' 12-month implementation of the four structural components of FAN (7, 8). In a sample of 1,308 church members (811 from intervention and 497 from control churches), members from intervention churches reported significantly greater implementation then members from control churches of PA opportunities, PA and HE messages, and pastor support for PA and HE at 12-months, with implementation of HE opportunities approaching statistical significance (7). The magnitude of these differences was large, ranging from d = 0.96 to 1.22. Consistent with member reports, church coordinators from intervention churches also reported significantly greater changes from baseline to 12 months in implementation than church coordinators from control churches for PA opportunities, PA and HE messages, pastor support for PA and HE, and guidelines for PA and HE (8). The magnitude of differences in these changes ranged from d=0.50 to 1.60 (except for opportunities for vegetables which did not differ over time by group as both groups scored high at baseline).

#### Effectiveness

Finally, the effectiveness of the intervention on member outcomes has been reported (7). Surveys conducted with members of intervention (*n* = 811) and control churches (*n* = 497) revealed that significantly fewer members of intervention churches were inactive as compared to members of control churches at 12 months. Fruit and vegetable intake, PA self-efficacy, and HE self-efficacy were also higher in members from intervention churches, although these differences were not statistically significant, but were similar in magnitude to results from our earlier and larger effectiveness trial (17).

### Physical Activity Maintenance

As shown in Table 1, statistically significant time effects were found for all PA components and for the implementation composite score. Church coordinators reported significantly greater implementation at 12 months compared to baseline for all PA components. They also reported significantly greater implementation at 24 months compared to baseline for the PA composite score, messages, opportunities, and pastor support. For PA guidelines (policies), the increase from baseline to 24 months approached statistical significance (*p* = 0.05). Scores at 24 months were significantly lower than scores at 12 months for all PA components. Effect sizes across components ranged from 1.18 to 2.04 from baseline to 12 months (large changes) and 0.43 to 1.02 from baseline to 24 months (medium to large changes).

**Table 1:** Change in physical activity and healthy eating implementation from baseline to 12 months and baseline to 24 months (*N* = 39 churches).

	**LSM (SE)**				**Effect size (d)**		***p*** **values**			
	**BL**	**12 M**	**24 M**	**BL SD**	**BL-12 M**	**BL-24 M**	**Time**	**BL-12 M**	**BL-24 M**	**12–24 M**
**PHYSICAL ACTIVITY**										
Composite score	1.70 (0.10)	2.77 (0.10)	2.24 (0.11)	0.52	2.04	1.02	<0.0001	<0.0001	<0.0001	<0.0001
Guidelines	1.95 (0.16)	2.83 (0.17)	2.27 (0.17)	0.75	1.18	0.43	<0.0001	<0.0001	0.0539	0.0012
Opportunities	1.81 (0.10)	2.71 (0.11)	2.31 (0.11)	0.66	1.35	0.75	<0.0001	<0.0001	0.0002	0.0024
Pastor support	1.49 (0.14)	2.84 (0.14)	2.24 (0.15)	0.76	1.79	0.99	<0.0001	<0.0001	<0.0001	0.0009
Messages	1.59 (0.12)	2.70 (0.12)	2.12 (0.12)	0.72	1.54	0.74	<0.0001	<0.0001	<0.0001	<0.0001
**HEALTHY EATING**										
Composite score	2.30 (0.09)	3.15 (0.09)	2.72 (0.09)	0.55	1.56	0.77	<0.0001	<0.0001	<0.0001	<0.0001
Guidelines	2.27 (0.16)	3.23 (0.17)	2.83 (0.17)	0.87	1.10	0.65	<0.0001	<0.0001	0.0014	0.0252
Opportunities	3.31 (0.11)	3.59 (0.11)	3.56 (0.11)	0.71	0.40	0.35	<0.0001	0.0003	0.0016	0.6774
Pastor support	1.82 (0.14)	2.87 (0.15)	2.18 (0.15)	0.90	1.17	0.40	<0.0001	<0.0001	0.0441	0.0003
Messages	1.79 (0.12)	2.86 (0.12)	2.28 (0.12)	0.78	1.37	0.63	<0.0001	<0.0001	0.0003	<0.0001

*LSM, least square mean; SE, standard error; BL, baseline; 12 M, 12 months; 24 M, 24 months; SD, standard deviation*.

*Results are from a repeated measures analysis. Possible scores for each area of implementation can range from 1 to 4, with 4 indicating greater implementation. Cohen's d calculated as 12-month (24-month) least square mean minus baseline least square mean divided by baseline standard deviation*.

Table 2 presents the PA implementation scores, by church, at the three time points, and their maintenance classification. A total of 42% of churches were classified as maintainers for guidelines (policies), 21% for opportunities, 36% for pastor support, and 21% for messages. Non-sustained implementers made up 24% of churches for guidelines (policies), 18% of churches for opportunities, 39% of churches for pastor support, and 24% of churches for messages. Finally, low implementers made up 33% of churches for guidelines (policies), 61% for opportunities, 24% for pastor support, and 55% for messages.

**Table 2:** Baseline, 12 and 24-month implementation scores and church categorization of 24-month maintenance status for physical activity components.

	**Guidelines**				**Opportunities**				**Pastor Support**				**Messages**			
**Church**	**BL**	**12 M**	**24 M**	**Cat**	**BL**	**12 M**	**24 M**	**Cat**	**BL**	**12 M**	**24 M**	**Cat**	**BL**	**12 M**	**24 M**	**Cat**
A	2.00	**3.00**			1.75	2.50			1.00	2.00			1.00	1.75		
B	2.00	**3.00**	**3.00**	M	1.75	2.50	2.25	LI	2.00	**3.00**	2.00	NSI	1.50	2.50	2.00	LI
C	1.00	2.00	1.00	LI	1.75	1.50	1.50	LI	1.00	**3.00**	1.00	NSI	1.00	1.75	1.00	LI
D	3.00	**4.00**			1.75	**3.25**			4.00	**4.00**			1.25	**3.50**		
E	2.00	**4.00**	2.00	NSI	3.75	**3.25**	**3.50**	M	1.00	**3.00**	1.00	NSI	3.25	**3.50**	**3.25**	M
F	2.00	**3.00**	1.00	NSI	2.00	2.00	1.50	LI	1.00	**3.00**	**3.00**	M	1.50	2.25	1.50	LI
G	2.00	2.00	1.00	LI	1.50	**3.50**	1.00	NSI	1.00	1.00	1.00	LI	1.25	2.50	1.50	LI
H	3.00	**4.00**	**3.00**	M	2.25	2.75	2.50	LI	3.00	2.00	2.00	LI	1.75	2.50	2.00	LI
I	2.00	**3.00**	2.00	NSI	1.25	1.75	1.50	LI	1.00	2.00	1.00	LI	1.00	1.50	1.00	LI
J	3.00	**3.00**	1.00	NSI	1.50	2.75	1.50	LI	1.00	**3.00**	1.00	NSI	1.00	2.50	1.00	LI
K	2.00	**3.00**	2.00	NSI	2.00	**3.00**	1.75	NSI	1.00	**3.00**	**3.00**	M	2.33	2.50	2.25	LI
L	1.00	1.00	1.00	LI	1.50	2.75	2.75	LI	1.00	**3.00**	1.00	NSI	1.50	**3.00**	1.50	NSI
M	3.00	**4.00**	**4.00**	M	1.50	**3.50**	**3.50**	M	2.00	**3.00**	**3.00**	M	1.00	**3.00**	2.75	NSI
N	2.00	1.00	2.00	LI	2.75	2.25	2.00	LI	2.00	**3.00**	2.00	NSI	1.50	2.75	1.75	LI
O	2.00	**3.00**	**4.00**	M	2.00	2.25	2.50	LI	2.00	**3.00**	2.00	NSI	1.50	2.75	1.50	LI
P	1.00		1.00	LI	1.75	2.25	2.00	LI	1.00	1.00	1.00	LI	1.00	**3.25**	1.00	NSI
Q	3.00	**4.00**	**4.00**	M	1.50	**3.25**	**3.25**	M	1.00	**3.00**	**4.00**	M	3.00	**3.25**	**4.00**	M
R	1.00	1.00	1.00	LI	1.25	2.75	1.00	LI	2.00	**3.00**	1.00	NSI	1.75	2.75	1.50	LI
S		**4.00**	**4.00**	M	3.00	**3.25**	2.50	NSI	1.00	**3.00**	**4.00**	M	3.00	2.75	2.50	LI
T	2.00	**4.00**	**3.00**	M	2.50	2.75	**3.00**	M	2.00	**3.00**	**3.00**	M	2.75	**3.00**	**3.50**	M
U	3.00	**3.00**	**4.00**	M	2.75	**3.50**	**3.50**	M	1.00	**4.00**	**4.00**	M	3.00	**3.25**	**3.25**	M
V	2.00	1.00	2.00	LI	1.00	2.25	2.00	LI	1.00	2.00	2.00	LI	1.50	1.75	1.00	LI
W	1.00	2.00	**3.00**	M	1.50	**3.00**	**3.75**	M	1.00	**4.00**	**3.00**	M	1.00	2.75	**3.00**	M
X	3.00	**4.00**	**3.00**	M	1.00	**3.25**	2.50	NSI	3.00	**3.00**	**3.00**	M	1.50	**3.00**	2.50	NSI
Y	1.00	**4.00**	1.00	NSI	2.00	2.67	2.50	LI	2.00	**3.00**	**4.00**	M	1.75	2.25	**3.25**	M
Z	3.00	**4.00**	**3.00**	M	3.00	2.67	2.50	LI	2.00	**4.00**	2.00	NSI	3.00	**3.25**	2.75	NSI
AA	1.00	2.00	2.00	LI	1.75	2.50	2.00	LI	1.00	**3.00**	2.00	NSI	1.00	**3.00**	1.75	NSI
BB	2.00	1.00	1.00	LI	1.25	2.00	2.25	LI	1.00	**3.00**	2.00	NSI	1.00	2.75	1.50	LI
CC	2.00	**4.00**	**4.00**	M	1.50	**3.25**	**3.50**	M	1.00	**3.00**	**4.00**	M	1.25	**3.25**	**4.00**	M
DD	2.00	**3.00**	**3.00**	M	1.75	**3.00**	2.75	NSI	1.00	2.00	2.00	LI	1.25	**3.25**	2.75	NSI
EE	1.00	**4.00**	**3.00**	M	1.00	2.75	2.00	LI	1.00	**3.00**	2.00	NSI	1.00	2.75	2.25	LI
FF	2.00	**3.00**	2.00	NSI	1.50	**3.00**	2.00	NSI	1.00	**3.00**	1.00	NSI	1.00	2.25	1.75	LI
GG	2.00	**3.00**	2.00	NSI	1.00	2.25	1.50	LI	1.00	2.00	1.00	LI	1.00	1.75	1.00	LI
HH	2.00	2.00	1.00	LI	2.50	2.50	2.25	LI	2.00	**4.00**	**3.00**	M	1.25	**3.25**	2.25	NSI
JJ	1.00	2.00	1.00	LI	1.25	2.50	1.75	LI	1.00	2.00	2.00	LI	1.00	2.75	1.75	LI

*BL, baseline; 12 M, 12-month assessment; 24 M, 24-month assessment; Cat, maintenance category*.

*Boldface indicates church met criteria for implementation or maintenance categorization. Maintainers (M) scored 3+ at 24 M. Non-Sustained Implementers (NSI) scored 3+ at 12 M but below 3 for 24 M maintenance. Low Implementers (LI) scored below 3 at 12 and 24 M*.

Table 3 presents the percentage of churches who met criteria for maintenance as well as the percentage of churches who either met criteria or showed improvements relative to baseline on four, three, two, one, and zero components of PA. Fifteen percent of churches were classified as maintainers on all four components, 3% on three components, 12% on two components, 27% on one component, and 42% on none of the components. Finally, 30% of churches met criteria or maintenance or improved relative to baseline on four components, 15% on three components, 18% on two components, 27% on one component, and 9% on none of the components.

**Table 3:** The number and percentage of churches that met criteria for maintenance and the percentage of churches that either met criteria for maintenance or showed an improvement at 24 months relative to baseline, by number of FAN physical activity components.

	**Met criteria for maintenance**		**Met criteria for maintenance or showed an improvement from baseline to 24 months**	
**Number of FAN physical activity components**	**Churches,** ***n***	**Churches,** **%**	**Churches,** ***n***	**Churches,** **%**
4	5	15.2	10	30.3
3	1	3.0	5	15.2
2	4	12.1	6	18.2
1	9	27.3	9	27.3
0	14	42.4	3	9.1

### Healthy Eating Maintenance

Statistically significant time effects were found for all HE components and for the HE composite score (Table 1). Church coordinators reported significantly greater implementation at 12 and 24 months compared to baseline, indicating that at 24 months, they were significantly above baseline levels. While these increases were sustained from 12 to 24 months for the HE opportunities, scores at 24 months were significantly lower than scores at 12 months for the other HE components and for the HE composite score. Effect sizes ranged from 0.40 to 1.56 from baseline to 12 months (medium to large changes) and 0.35–0.77 from baseline to 24 months (small to medium changes).

As shown in Table 4, 52% of churches were classified as maintainers for HE guidelines (policies), 94% for opportunities, 36% for pastor support, and 27% for messages. Non-sustained implementers made up 30% of churches for guidelines (policies), 3% of churches for opportunities, 42% of churches for pastor support, and 33% of churches for messages. Finally, low implementers made up 18% of churches for guidelines (policies), 3% for opportunities, 21% for pastor support, and 39% for messages.

**Table 4:** Baseline, 12 and 24-month implementation scores and church categorization of 24-month maintenance status for healthy eating components.

	**Guidelines**				**Opportunities**				**Pastor Support**				**Messages**			
**Church**	**BL**	**12 M**	**24 M**	**Cat**	**BL**	**12 M**	**24 M**	**Cat**	**BL**	**12 M**	**24 M**	**Cat**	**BL**	**12 M**	**24 M**	**Cat**
A	2.00	**4.00**			3.50	**4.00**			1.00	2.00			1.25	2.50		
B	2.00	**3.00**	**3.00**	M	3.00	**3.50**	**3.00**	M	2.00	**3.00**	2.00	NSI	1.50	**3.00**	2.00	NSI
C	3.00	2.00	**3.00**	M	3.50	**3.50**	**4.00**	M	2.00	**3.00**	2.00	NSI	1.25	2.75	1.25	LI
D	2.50	**4.00**			4.00	**4.00**			1.00	**3.00**			1.25	**3.00**		
E	2.00	**3.00**	2.00	NSI	3.50	**3.00**	**3.00**	M	1.00	**3.00**	1.00	NSI	3.25	**3.50**	**3.00**	M
F	2.00	**4.00**	**4.00**	M	3.50	**4.00**	**4.00**	M	2.00	**3.00**	**3.00**	M	1.50	**3.00**	2.00	NSI
G	1.50	**3.50**	1.00	NSI	3.00	**3.50**	**4.00**	M	1.00	**3.00**	1.00	NSI	1.50	2.50	1.50	LI
H	2.00	**4.00**	2.50	NSI	3.00	**4.00**	**4.00**	M	2.00	2.00	**3.00**	M	2.50	2.75	2.75	MLI
I	2.50	**3.00**	2.50	NSI	2.00	**3.00**	**3.00**	M	1.00	2.00	2.00	LI	1.25	1.25	1.25	LI
J	3.00	**4.00**	**4.00**	M	3.50	**4.00**	**4.00**	M	2.00	**4.00**	1.00	NSI	1.00	2.75	1.00	LI
K	2.00	**3.00**	2.00	NSI	3.50	**3.00**	**3.00**	M	1.00	**4.00**	1.00	NSI	2.25	2.75	2.25	LI
L	1.00	1.00	1.00	LI	3.50	**4.00**	**3.50**	M	1.00	**3.00**	1.00	NSI	2.00	**3.25**	2.25	NSI
M	2.50	**4.00**	**4.00**	M	3.50	**4.00**	**4.00**	M	3.00	**3.00**	2.00	NSI	2.25	**3.25**	2.50	NSI
N	2.00	1.00	1.50	LI	4.00	**4.00**	**3.50**	M	2.00	**3.00**	1.00	NSI	1.75	2.75	2.25	LI
O	2.00	**3.50**	**4.00**	M	3.00	**3.00**	2.50	NSI	3.00	**3.00**	2.00	NSI	1.75	2.75	1.50	LI
P	1.00	**4.00**	**3.50**	M	3.50	**3.50**	**3.50**	M	1.00	**4.00**	1.00	NSI	1.00	**3.75**	2.00	NSI
Q	4.00	**4.00**	**4.00**	M	4.00	**4.00**	**4.00**	M	3.00	**3.00**	**4.00**	M	3.50	**3.25**	**4.00**	M
R	3.00	**3.50**	2.00	NSI	4.00	**3.50**	**4.00**	M	2.00	2.00	2.00	LI	2.75	2.50	2.50	LI
S	4.00	**4.00**	**4.00**	M	4.00	**4.00**	**4.00**	M	1.00	**3.00**	**3.00**	M	3.25	2.25	**3.00**	M
T	2.50	**4.00**	**4.00**	M	3.00	**4.00**	**4.00**	M	2.00	**3.00**	**3.00**	M	3.00	**3.50**	**3.50**	M
U	3.50	**4.00**	**4.00**	M	3.50	**3.50**	**3.50**	M	2.00	**4.00**	**4.00**	M	3.00	**3.50**	**3.00**	M
V	2.00	1.00	1.50	LI	3.00	**4.00**	**3.50**	M	1.00	2.00	1.00	LI	1.25	1.75	1.50	LI
W	1.00	**4.00**	**4.00**	M	3.50	**4.00**	**4.00**	M	2.00	**3.00**	**4.00**	M	1.00	2.25	**3.25**	M
X	2.50	**3.50**	**4.00**	M	4.00	**4.00**	**4.00**	M	3.00	**3.00**	**3.00**	M	2.25	**3.00**	2.25	NSI
Y	1.00	1.00	1.00	LI	3.50	**4.00**	**4.00**	M	1.00	1.00	2.00	LI	1.00	2.00	1.75	LI
Z	3.50	**4.00**	**3.50**	M	3.00	**3.00**	**3.00**	M	3.00	**4.00**	**3.00**	M	3.25	**3.50**	**3.25**	M
AA	1.50	2.00	2.00	LI	4.00	**4.00**	**4.00**	M	1.00	2.00	2.00	LI	1.25	**3.25**	2.00	NSI
BB	2.00		2.50	LI	4.00	**4.00**	**4.00**	M		**3.00**	2.00	NSI	1.33	**3.67**	1.50	NSI
CC	2.00	**4.00**	**4.00**	M	3.00	**3.50**	**4.00**	M	1.00	**3.00**	**3.00**	M	1.75	2.75	**3.75**	M
DD	2.50	**3.50**	**4.00**	M	2.50	**3.50**	**4.00**	M	1.00	2.00	**3.00**	M	1.00	**3.50**	**3.00**	M
EE	3.00	**4.00**	2.50	NSI	3.00	**4.00**	**3.00**	M	1.00	**3.00**	2.00	NSI	1.25	**3.25**	1.75	NSI
FF	3.00	**4.00**	**4.00**	M	4.00	**4.00**	**4.00**	M	2.00	**3.00**	1.00	NSI	1.25	2.25	1.50	LI
GG	2.00	**3.00**	2.50	NSI	1.50	1.00	1.50	LI	1.00	2.00	1.00	LI	1.00	2.00	1.50	LI
HH	4.00	**4.00**	2.00	NSI	3.50	**4.00**	**3.50**	M	3.00	**3.00**	**3.00**	M	1.50	**3.00**	2.75	NSI
JJ	2.50	**3.00**	1.00	NSI	3.50	**3.50**	**3.50**	M	1.00	2.00	2.00	LI	1.00	**3.50**	2.50	NSI

*BL, baseline; 12 M, 12-month assessment; 24 M, 24-month assessment; Cat, maintenance category*.

*Boldface indicates church met criteria for implementation or maintenance categorization. Maintainers (M) scored 3+ at 24 M. Non-Sustained Implementers (NSI) scored 3+ at 12 M but below 3 for 24 M maintenance. Low Implementers (LI) scored below 3 at 12 and 24 M*.

Table 5 presents the percentage of churches who met criteria for maintenance as well as the percentage of churches who either met criteria or showed improvements relative to baseline on four, three, two, one, and zero components of HE. Twenty-four percent of churches were classified as maintainers on all four components, 6% on three components, 27% on two components, 39% on one component, and 3% on none of the components. Finally, 33% of churches met criteria or maintenance or improved relative to baseline on four components, 30% on three components, 24% on two components, 12% on one component, and 0% on none of the components.

**Table 5:** The number and percentage of churches that met criteria for maintenance and the percentage of churches that either met criteria for maintenance or showed an improvement at 24 months relative to baseline, by number of FAN healthy eating components.

	**Met criteria for maintenance**		**Met criteria for maintenance or showed an improvement from baseline to 24 months**	
**Number of FAN healthy eating components**	**Churches,** ***n***	**Churches,** **%**	**Churches,** ***n***	**Churches,** **%**
4	8	24.2	11	33.3
3	2	6.1	10	30.0
2	9	27.2	8	24.2
1	13	39.4	4	12.1
0	1	3.0	0	0.0

## Discussion

This study's focus on organizational change and sustainability contributes to the faith-based (and other organizational) interventions literature, as well as to dissemination and implementation research and process evaluation literatures. Our focus on organizational change, consistent with the structural model of health behavior, (15) rather than on individual behavior change, makes FAN distinct in the faith-based literature (18–20). The intervention, developed using a community-based participatory research approach, (14) was designed to increase church capacity, with the goal of fostering sustainable changes in the church setting.

While there are examples of faith-based interventions that are based on ecological models, (21–26) the policy, systems, and environmental changes are rarely a central focus of the intervention, and organizational outcomes are measured infrequently. Even fewer faith-based interventions have examined program sustainability. In the North Carolina Black Churches United for Better Health Study (49 churches), (25) member behavior change (fruit and vegetable intake) was found to be maintained over a 2-year period in intervention vs. control churches, and although organizational maintenance was targeted in the intervention, it was not systematically examined.

The FAN D&I study used the RE-AIM framework to study adoption through maintenance. RE-AIM has been applied to a variety of topic areas and settings. Literature reviews consistently conclude that organizational maintenance is reported at lower levels than the other dimensions of RE-AIM. For example, Antikainen et al. (27) found that among theory-based PA intervention studies, organizational maintenance was reported in only 5% (*n* = 3) of studies. The reporting of organizational maintenance in childhood and youth PA, diet, and obesity studies has also been low (28–31). Harden et al.'s systematic review of behavioral interventions found that there was insufficient data to determine the average organizational maintenance for the 82 interventions included (32). A recent 20-year review of studies using RE-AIM concluded that there are limited data on outcomes after interventions end (33). Our study not only reported organizational maintenance using distinct criteria, but also reported the maintenance of each intervention component using a continuous scale, avoiding the “all or nothing” view of maintenance, as recommended by Scheirer et al. (34).

Our findings are encouraging. This paper summarized the positive and meaningful findings previously reported for reach, adoption, implementation, and effectiveness in this study. With regard to maintenance, although mean implementation scores of the majority of PA and HE components decreased from 12 to 24 months, churches had significantly higher implementation at 24 months than they did at baseline, with effect sizes ranging from *d* = 0.35 to 1.02 (most were medium to large). Furthermore, 21–42% of churches were classified as maintainers across the four PA components, and 27–94% across the HE components. Fifteen percent of churches were classified as maintainers on all four PA components, and 24% were classified as maintainers on all four healthy heating components. Most churches maintained at least one FAN component. Furthermore, when considering the percentage of churches that were either classified as maintainers or showed an improvement from baseline to 24 months, 30% of churches did so for all four PA components and 33% for all four HE components, with the majority of churches showing improvements on at least two of the PA and HE components.

Several factors likely contributed to sustained improvements relative to baseline at 24 months. First, FAN is a flexible program organized around core elements rather than a rigid curriculum. Churches were enabled to select activities from within the conceptual model that fit their church culture, demographics, customs, and resources. Second, the intervention was developed in partnership with church leaders and lay members using a community-based participatory research approach, was consistent with the idea of designing for dissemination (35), and resulted in a good fit with church practices and beliefs. Third, churches received a year of technical assistance telephone calls to help support program implementation (9) which has been shown to be important for organizations implementing evidence-based programs (36). Community health advisors, and not research staff, delivered the training and technical assistance to churches, resulting in meaningful changes in member and organizational outcomes (7, 8), and an approach that is likely to be more cost-effective.

For PA, using categorical outcomes, the greatest maintenance was of guideline (policy) changes and pastor support for PA. Maintenance of opportunities and messages were the lowest, likely because fewer than half of the churches met criteria for high implementation at 12 months for these two components. In contrast, for HE, maintenance of opportunities was the highest, with most churches providing members fruit and vegetables when food was served. HE policies was the second most frequently maintained component, with maintenance of pastor support of PA and HE messages the lowest. An earlier analysis of qualitative data from our study showed that resistance to change was among the most common barriers to implementation that pastors and church coordinators reported, whereas leader support was an important enabler (10). Because many churches provide meals and snacks to members, it might have been easier to make modifications to an existing practice rather than add a completely new activity (PA).

It was also notable that the percentage of churches categorized as “non-sustained implementers” was relatively high for some of the components of FAN. These were churches that met criteria for high implementation at 12 months, but no longer met the criteria at 24 months. For example, across the PA components, these percentages ranged from 18% (opportunities) to 39% (pastor support), and across the HE components, these percentages ranged from 3% (opportunities) to 42% (pastor support). It might be that the criteria for implementation were too stringent for some components and perhaps not realistic in the faith-based setting whose main focus is on the spiritual well-being of members. For example, it is probably not realistic that pastors include messages about PA and HE at least monthly or that the church distribute PA and HE materials at this frequency over time. Rather, it might be more important and realistic for members to hear and see messages frequently for a shorter period of time and then continue to hear and see messages over time, but less frequently, to reinforce the importance of PA and HE. Adjusting the threshold of acceptable implementation would have yielded substantially more favorable maintenance findings, and our future projects will reconsider what constitutes both meaningful and realistic implementation. However, we opted to use our a prior definition for 12-month implementation and apply this same criteria at 24-months for this paper. FAN's focus is on organizational change which is challenging and may require more time and technical assistance to produce and maintain relative to individual level change. Identifying which churches are more likely to need greater assistance is an important next step for research in this area.

There are several limitations to this study. First, we were reliant on church coordinator reports of implementation at all time points and were unable to do on-site observations. Although we cannot rule out social desirability biases, our implementation data as reported by church coordinators and perceived by members has been congruent, both in this Phase of the study (7, 8) and in a previous study of FAN (16, 17). Second, we did not collect in-depth information regarding the specific policy, systems, and environmental changes made, as was done by Boutain et al. (37). Future studies should focus on the factors that enabled some churches to successfully maintain all or parts of FAN. Third, we did not have a control group for comparison for maintenance. Our study design used a delayed-intervention control group, and the control group was trained 1 year after the intervention group. As reported in earlier papers, (7, 8) we demonstrated differences between intervention and control churches on implementation and effectiveness outcomes at 12 months, but we could not make these comparison for maintenance, as we did not have a control group unexposed to the intervention for 24 months.

This study also has several strengths. First, it is one of the largest faith-based interventions conducted to date (59 churches randomized; 39 in the intervention group). Only 4 of the 39 intervention churches did not participate in subsequent interviews. Church coordinator interview completion was high at 12 (35/39) and 24 months (33/39). We assessed implementation at three time points (baseline, 12, 24 months), allowing us to examine patterns over time. Furthermore, it is the only study, to our knowledge, to assess organizational maintenance in this setting. Finally, this study is unique in its reporting of each component of the RE-AIM framework.

## Conclusion

In this county-wide dissemination of an evidence-based program, delivered to churches by community health advisors, we found that PA and HE implementation increased significantly from baseline to 12 months and from baseline to 24 months, and these increases were medium to large in magnitude. We also found that most churches maintained at least one component of FAN at 24 months. These promising findings position FAN well for the national implementation study now underway.

## Data Availability Statement

The datasets generated for this study will not be made publicly available. The dataset generated and analyzed during the current study is not publicly available due to concerns about confidentiality with the limited number of participating churches in a specific geographical region. Requests for data and data analyses will be considered and honored if they do not duplicate work conducted or underway by our team and appear reasonable. Requests to access the datasets should be directed to SW, wilcoxs@mailbox.sc.edu.

## Ethics Statement

The study involved human participants and was reviewed and approved by the University of South Carolina Institutional Review Board and was granted exempt status. Written informed consent for participation was not required for this study in accordance with the national legislation and the institutional requirements.

## Author Contributions

SW, RS, and BH were involved in writing the grant proposal and conceptualizing the study. DJ-S participated in enrolling churches. SW, RS, and DJ-S selected study measures and developed the church visit protocol. SW was involved in making day-to-day decisions on the project, with community input. BH and SW conducted data analyses. SW drafted the manuscript. All authors substantially reviewed and edited the manuscript.

## Conflict of Interest

SW, RS, and BH were supported during the preparation of this article by a cooperative agreement from the Centers for Disease Control and Prevention (U48DP005000). The remaining author declares that the research was conducted in the absence of any commercial or financial relationships that could be construed as a potential conflict of interest.
